# Supplementary material for: Genetic Diversity and Spatiotemporal Distribution of SARS-CoV-2 Variants in Guinea: A Meta-Analysis of Sequence Data (2020–2023)
Source: Viruses. 2025 Jan 31;17(2):204. doi: 10.3390/v17020204 (PMC11860642; doi:10.3390/v17020204)
Supplement: Supplementary file 1 [file viruses-17-00204-s001.zip › viruses-3398464-supplementary.pdf]

Supplementary Table S1: Number of cases and the number of hospital deaths by the major waves and dominant variants

Major wave	Dominant variants	Periode	Number of cases N(%)	Number of hospital deaths N(%)
**1st Wave**	B.1<br>B.1.1	March 2020 to January 2021	14 532 (37,6)	82 (17,5)
**2nd Wave**	Alpha (B.1.1.7)<br>Eta (B.1.525)<br>B.1.629<br>R.1	February 2021 to Jun 2021	9 160 (23,7)	86 (18,3)
**3rd Wave**	Delta (B.1.617.2)	Jult 2021 to November 2021	7 071 (18,3)	219 (46,7)
**4th Wave**	Omicron (BA.1)	December 2021 to March 2022	5 696 (14,7)	53 (11,3)
**No significant waves**	Omicron (BA.2. BA.5, BQ.1, XBB.1)	April 2022 to December 2023	2 113 (5,4)	28 (5,9)
		**Total**	**38572**	**468**

No significant waves: During this period, we noted many small peaks marked by the circulation of Omicron sublineages

Supplementary Table S2: Variants analyzed and their sublineages included in the datasets for Guinea sequences used in the phylogenetic and phylogeography analysis

Variant (WHO Name)	Sublineages
**B.1.1.7 (Alpha)**	-
**Eta (B.1.525)**	-
**B.1.617.2 (Delta)**	AY.122; AY.126; AY.34; AY.34.1; AY.36; AY.37; AY.6; AY.98
**Omicron BA.1**	BA.1.1; BA.1.1.10 ;BA.1.13; BA.1.14; BA.1.15.1; BA.1.16
**Omicron BA.2**	BA.2; BA.2.1; BA2.2; BA2.3; BA2.4; BA2.5; BA2.7; BA2.8; BA2.9
**Omicron BA.5**	BA.5.1; BA.5.2; BA.5.2.1; BA.5.1.22; BA.5.2.25; BA.5.5
**Omicron BQ.1**	BQ.1.1; BQ.1.25
**Omicron XBB.1**	XBB.1.17.1; XBB.1.5; XBB.1.5.4; XBB.1.5.58; XBB.1.5.89
